# Evolution of short inverted repeat in cupressophytes, transfer of *accD* to nucleus in *Sciadopitys verticillata* and phylogenetic position of Sciadopityaceae

**DOI:** 10.1038/srep20934

**Published:** 2016-02-11

**Authors:** Jia Li, Lei Gao, Shanshan Chen, Ke Tao, Yingjuan Su, Ting Wang

**Affiliations:** 1 CAS Key Laboratory of Plant Germplasm Enhancement and Specialty Agriculture, Wuhan Botanical Garden, Chinese Academy of Sciences, Wuhan, Hubei, China; 2 University of Chinese Academy of Sciences, Beijing, China; 3 State Key Laboratory of Biocontrol, School of Life Sciences, Sun Yat-sen University, Guangzhou, Guangdong, China; 4 Institute for Technology Research and Innovation of Sun Yat-sen University, Zhuhai, Guangdong, China; 5 College of Life Sciences, South China Agricultural University, Guangzhou, Guangdong, China

## Abstract

*Sciadopitys verticillata* is an evergreen conifer and an economically valuable tree used in construction, which is the only member of the family Sciadopityaceae. Acquisition of the *S. verticillata* chloroplast (cp) genome will be useful for understanding the evolutionary mechanism of conifers and phylogenetic relationships among gymnosperm. In this study, we have first reported the complete chloroplast genome of *S. verticillata*. The total genome is 138,284 bp in length, consisting of 118 unique genes. The *S. verticillata* cp genome has lost one copy of the canonical inverted repeats and shown distinctive genomic structure comparing with other cupressophytes. Fifty-three simple sequence repeat loci and 18 forward tandem repeats were identified in the *S. verticillata* cp genome. According to the rearrangement of cupressophyte cp genome, we proposed one mechanism for the formation of inverted repeat: tandem repeat occured first, then rearrangement divided the tandem repeat into inverted repeats located at different regions. Phylogenetic estimates inferred from 59-gene sequences and cpDNA organizations have both shown that *S. verticillata* was sister to the clade consisting of Cupressaceae, Taxaceae, and Cephalotaxaceae. Moreover, *accD* gene was found to be lost in the *S. verticillata* cp genome, and a nucleus copy was identified from two transcriptome data.

Generally, seed plant chloroplast (cp) DNAs present a conserved quadripartite structure, with a pair of inverted repeats (IR) separating the whole genome into a large single copy (LSC) and small single copy region (SSC)^1^. The most significant feature of coniferous cp genomes is that one copy of the canonical rRNA-containing IR has been lost. However, evolution has endowed conifers cp genomes with short novel repeats^2^,^3^,^4^,^5^. These repeats can replace the function of canonical IR to promote homologous recombination (HR), thus generating isomeric genomic forms^3^,^5^,^6^, such as type 1 and 3 repeats in Pinaceae^3^ and *trnQ* repeats in *Cephalotaxus oliveri*^5^ and *Juniperus*^6^. There are no common short inverted repeats (sIRs) that are larger than 100 bp found within cupressophytes *per se*. Nevertheless, Cupressaceae, Taxaceae, and Cephalotaxaceae share a common sIR embracing *trnQ-UUG* (optional expanding to 5′ end of *chlB* in the genus *Cephalotaxus*), ranging from 230 bp in *Taxus mairei* to 544 bp in *C. oliveri*. In *C. oliveri*, the *trnQ-UUG* sIR was inferred to mediate HR, and two isomeric cpDNA forms were detected^5^. In *Taiwania cryptomerioides* and *Cryptomeria japonica*, the *trnQ-UUG* sIR is only approximately 280 bp, but not any alternative form was detected^5^. By contrast, in four *Juniperus* species, the substoichiometric presence of the rearranged isomeric form caused by the *trnQ-UUG* sIR was identified by using both PCR and high-throughput read-pair mapping method^6^. Comparative genomic studies demonstrated that the predominant and substoichiometric arrangements of the sIR have shifted several times during the cupressophyte evolution^6^. Considering the absence of the sIR in Araucariaceae and Podocarpaceae and its presence in other cupressophytes, the sIR was deduced to originate in the common ancestor of Cupressaceae, Cephalotaxaceae, and Taxaceae^6^. However, the underlying evolutionary mechanisms of the formation of sIR have not been well studied. More cupressophyte cpDNAs are needed to dissect the detailed pathway. To date, the cp genomic sequences of cupressophytes have been reported for 26 species from five families, i.e. Taxaceae^7^, Cephalotaxaceae^5^,^8^, Cupressaceae^9^, Araucariaceae^10^, and Podocarpaceae^11^. Thus Sciadopityaceae becomes the only one family in cupressophyte whose chloroplast genome has not been reported. In this study, we have sequenced the entire cpDNA of *Sciadopitys verticillata* and performed a comparative analysis of the cupressophyte cp genomes.

Currently, it is still controversial about the phylogenetic position of Sciadopityaceae. Most of the molecular phylogenetic studies supported that Sciadopityaceae is phylogenetically isolated of all conifers, branching off firstly from the Cupressaceae-Taxaceae-Cephalotaxaceae clade^12^,^13^. Few studies also insisted that Sciadopityaceae is sister to a clade comprising Podocarpaceae and Araucariaceae^14^. Chloroplast genome information can serve as useful markers for resolving phylogenetic relationships between Sciadopityaceae and other cupressophytes.

Chloroplast is considered to originate from cyanobacteria through ancient endosymbiosis^15^. In this process, many nonessential genes were lost, and some functional genes were relocated from chloroplast to the nuclear genome^16^. Four genes have been reported functionally transferred from the plastid to the nucleus: *infA* in multiple lineages, including most rosids^17^; *rpl22* in Fabaceae and Fagaceae^18^,^19^; *rpl32* in Rhizophoraceae and Salicaceae (two families of Malpighiales) and Thalictroideae^20^,^21^,^22^ and *accD* in Campanulaceae and Fabaceae^23^,^24^. Moreover, independent loss of *accD* has also been documented in the cp genomes of Acoraceae^25^, Poaceae^26^,^27^, Geraniaceae^28^, and Gnetales^29^. In Poaceae, the prokaryotic type acetyl-CoA carboxylase (ACCase) in the plastid has been completely replaced by a nucleus-encoded eukaryotic type ancestry^30^. However, it remains unknown whether the *accD* loss has occurred in the cp genome of other gymnosperms beside Gnetales. As Sciadopityaceae is the only one family whose cp genome has not been determined in gymnosperms, sequencing the cp genome of *Sciadopitys verticillata* will also provide useful information on this issue.

To further understand the evolution of cupressophyte cp genomes and elucidate the phylogenetic relationships among gymnosperms, we have 1) determined the complete cp genome of *S. verticillata*; 2) compared the overall gene content and organization of *S. verticillata* cp genome with those of other cupressophytes, detailing gene loss and its substitution mechanism; 3) detected repeat sequences in *S. verticillata* and further investigated the evolutionary mechanism of sIR in cupressophytes; and 4) examined the phylogenetic position of Sciadopityaceae among cupressophytes.

## Results

### General features of *S. verticillata* cp genome

**Figure 1:**
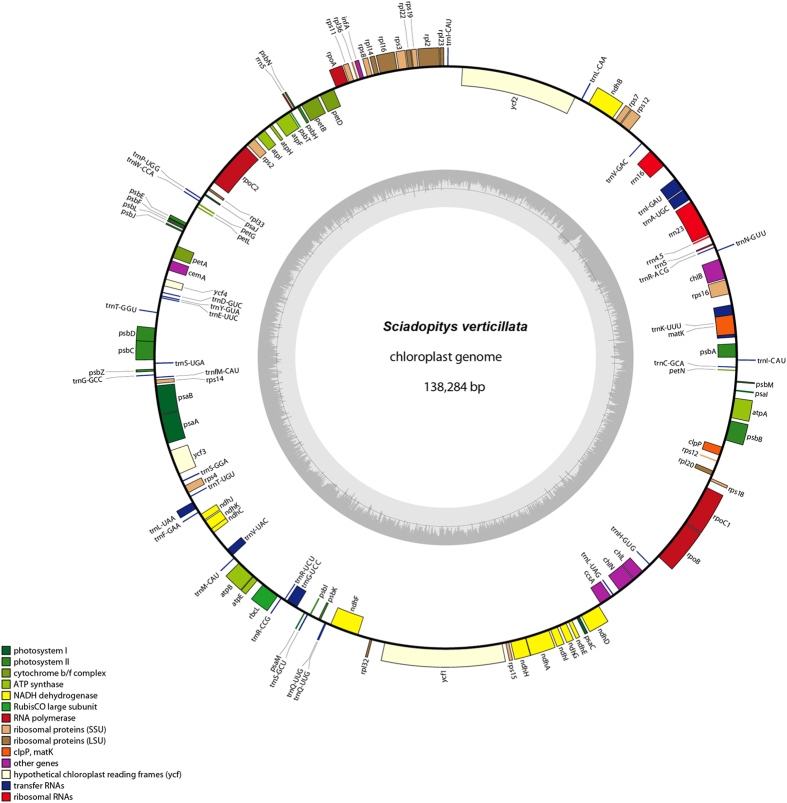
Gene map of the *Sciadopitys verticillata* plastid genome. Genes shown inside and outside the circle are transcribed clockwise and counterclockwise, respectively.

The complete cp genome of *S. verticillata* is a circular molecule of 138,284 bp (Fig. 1), only shorter than that of *Agathis dammara* (145,625 bp) in cupressophytes. Like other sequenced cp genomes of conifers, the *S. verticillata* cp genome does not contain one copy of canonical IR. The overall GC content is 35.42% (protein-coding genes, 36.35%; tRNA genes, 52.79%; rRNA genes, 52.45%; introns, 35.93%; intergenic spacers, 30.65%) (Table 1). In *S. verticillata* cp genome, 118 unique genes were identified, including 83 protein-coding genes, 31 tRNA genes and 4 rRNA genes. Among these 118 unique genes, *rrn5, trnI-CAU* and *trnQ-UUG* are duplicated. *Rrn5* occurred as an inverted repeat sequence, while two copies of *trnI-CAU* and *trnQ-UUG* arranged in the same orientation. Ten protein-coding genes and six tRNA genes each have one intron, while *ycf3* contains two. When comparing *S. verticillata* cpDNA with other conifers, the *trnP-GGG* and *accD* gene were lost. The *trnP-GGG* is also absent in *Ephedra equisetina*, whereas it is present as complete and functional in other conifers, *Cycas, Ginkgo, Gnetum* and *Welwitschia*^29^. The loss of *accD* was also found in Gnetales^29^. *AccD* gene encodes acetyl-CoA carboxylase β subunit and is essential in fatty acid synthesis^26^. At last, a C-to-U RNA editing site was identified at the initial codon of *rps8* and verified by cDNA sequencing.

### The repeat sequence

In this study, we have analyzed the occurrence, nature, organization, and distribution of simple sequence repeat (SSR) in the *S. verticillata* cp genome. In total, 53 SSRs were identified (Supplementary Table S1). Of them, 34 SSR loci are located within intergenic spacers (IGS), 6 in introns and 13 in coding regions. Three regions containing multiple SSRs (3 SSRs in *trnI-GAU* to *rrn16* IGS, 2 in *rps12* intron, 3 in *petA* to *cemA* IGS) are particular valuable for population genetics studies as they are co-located in short regions^31^. Among these SSRs, 36 are mononucleotide, 11 are dinucleotide, 5 are trinucleotide and only one is tetranucleotide. This suggests that the most common type of cp repeat is the mononucleotide, whereas di-, tri- or tetranucleotide repeats are rare.

Tandem repeat finder was used to identify the tandem repeats in *S. verticillata* cp genome, with the identity and size of the repeats were limited to no less than 90% and 30 bp in unit length. In total, 18 forward tandem repeats in *S. verticillata* cp genome were identified (Supplementary Table S2), of which 11 are located in coding regions of *ycf2* (6), *trnQ-UUG* (1), and *ycf1* (4). The other seven tandem repeats are distributed in the IGS of *trnI-GAU*/*rrn16* (2), *rrn16*/*trnV-GAC* (1), *ycf2*/*trnI-CAU* (1), *trnM-CAU*/*atpB* (1), *rpl32*/*ycf1* (1), and *rpoC1* intron (1). The cp genome of *S. verticillata* has 18 forward tandem repeats, similar to *C. oliveri* cp genome (17)^5^, but less than the cp genome of *Podocarpus lambertii* (28)^11^. The *ycf1* gene in the three genomes all contains tandem repeat. Interestingly, one tandem repeat containing *trnQ-UUG* gene was detected in *S. verticillata* cp genome, which exactly forming two copies of *trnQ-UUG* gene concatenated.

### The duplication of *trnQ-UUG* in *S. verticill*a*ta* cp genome

**Figure 2:**
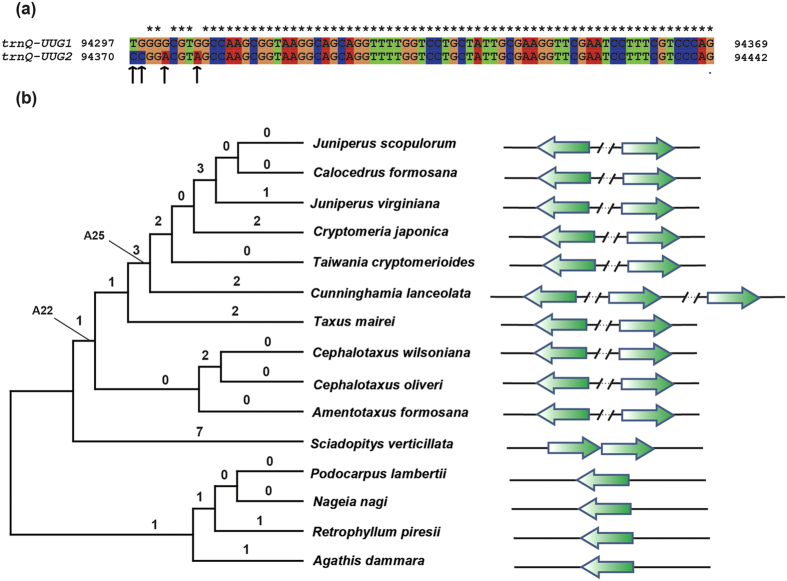
The evolution of *trnQ-UUG* in cupressophytes. (**a**) The alignment of two *trnQ-UUG* copies in *Sciadopitys verticillata* chloroplast genome. Four black arrows under the alignment denote the different nucleotide sites. The numbers in the left and right denote the relative position in the complete chloroplast genome. The two copies of *trnQ-UUG* formed a tandem repeat sequence. (**b**) The topology in the left was the phylogenetic tree of the cupressophyte inferred from the matrices of chloroplast DNA locally collinear blocks. The number of rearrangements leading to a clade was shown above the branch. A22 indicates the common ancestor of Cephalotaxaceae, Taxaceae, and Cupressaceae, and A25 indicates the common ancestor of Cupressaceae. The green arrows in the right denote *trnQ-UUG* gene. The direction of the arrows denotes the relative direction of *trnQ-UUG* gene. The regions between the two slashes indicate the omitted gene.

Although Sciadopityaceae cp genome does not contain *trnQ-UUG* sIR, two copies of *trnQ-UUG* were clustered as a tandem repeat (Fig. 2). Four nucleotide sites were different between the two copies of *trnQ-UUG* gene (Fig. 2a). Sciadopityaceae was the first species that started to contain two copies of *trnQ-UUG*. Podocarpaceae and Araucariaceae each have only one copy of *trnQ-UUG*. In order to understand the evolutionary process of *trnQ-UUG* in cupressophytes, the cp genome rearrangement and ancestral plastomic organization among cupressophytes were inferred. The permutation with 33 locally collinear blocks (LCBs) was generated on the basis of whole plastome alignments among the 15 cupressophyte species. The permutation was then used to construct the most parsimonious tree (Fig. 2b). The resulting tree has two major clades, one comprising Cephalotaxaceae, Taxaceae, Cupressaceae, and Sciadopityaceae which have the *trnQ-UUG* repeat, while the other including Podocarpaceae and Araucariaceae which does not contain *trnQ-UUG* repeat. The Sciadopityaceae containing the tandem repeat was located at the basal position within the first clade. So we speculate that the tandem repeat of *trnQ-UUG* came first, the inverted repeats may result from the evolution of rearrangement.

**Figure 3:**
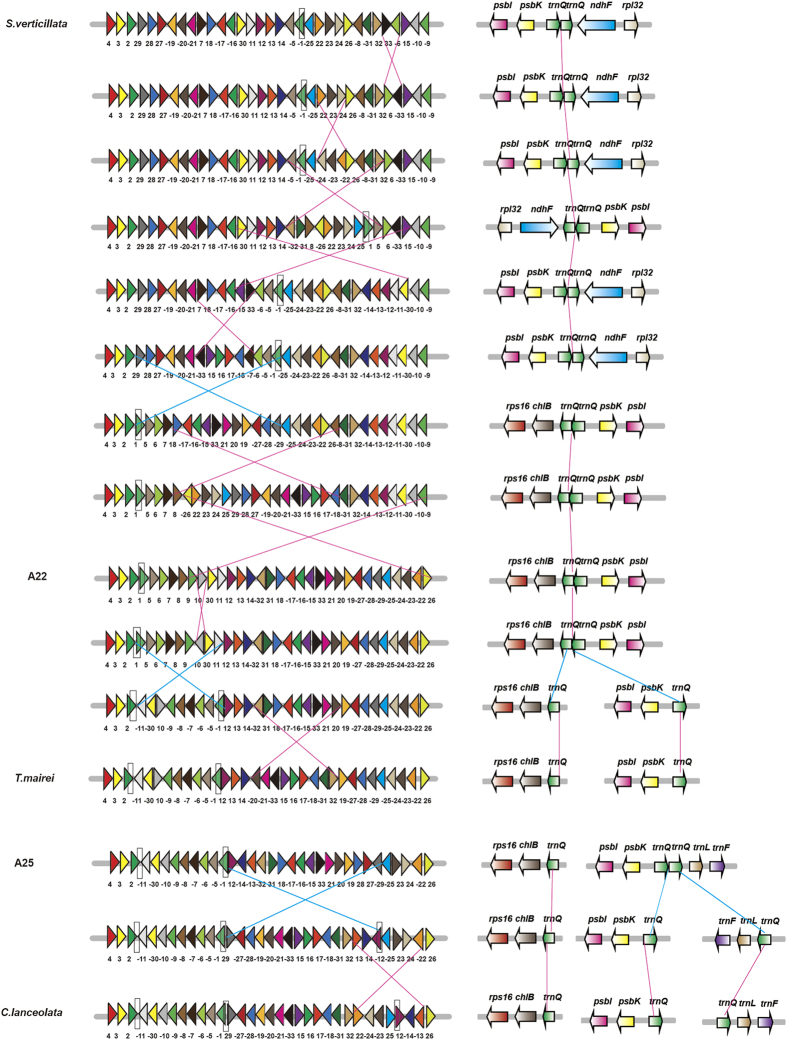
The formation mechanism of *trnQ-UUG* inverted repeat sequence along with the plastomic rearrangements in cupressophytes. Two hypothetical evolutionary scenarios for plastomic rearrangements were shown: from *Sciadopitys verticillata* to *Taxus mairei* and from A25 to *Cunninghamia lanceolata.* Plastomes are circular but here are shown in gray bars. Locally collinear blocks (LCBs) with their relative orientations were indicated with color triangles. Inversions between two plastomes were linked by carmine and blue lines. Three pairs of blue lines denote the inversion occurred between LCB 29 and 1, LCB 1 and 11, LCB 12 and 29. The locations of *trnQ-UUG* in the LCB were shown in the black box. The arrows in the right panel denote the specific location of *trnQ-UUG*. The direction of arrows denotes the orientations of the gene. Carmine and blue lines between *trnQ-UUG* genes denote the evolutionary scenarios along with plastomic rearrangements.

Figure 3 shows two detailed evolutionary scenarios of plastomic rearrangements: one from *S. verticillata* to *Taxus mairei* through A22 (the ancestral plastomic organization of Cupressaceae, Taxaceae, and Cephalotaxaceae), the other from A25 (the ancestral plastomic organization of Cupressaceae) to *Cunninghamia lanceolata*. Here, we propose an alternative mechanism for the formation of *trnQ-UUG* sIR following the rearrangement of cp genome. Two tandem *trnQ-UUG* copies are first located in the spacer region of LCB 1 and 25, which is between *psbI*-*psbK* and *ndhF*-*rpl32* gene (Fig. 3). From *S. verticillata* to A22, one inversion was happened between LCB 29 and 1, making LCB 1 adjacent to LCB 2 which contains *rps16* and *chlB* gene (Fig. 3 the blue line). During this process, the two copies of *trnQ-UUG* moved following the LCB 1, forming the *rps16*-*chlB*-*trnQ*-*trnQ*-*psbK*-*psbI* gene order (Fig. 3). From A22 to *Taxus mairei*, one inversion occurred between LCB 1 and 11 (Fig. 3 the blue line). During this inversion, one copy of *trnQ-UUG* gene followed LCB1, while the other followed the LCB 2. Then the tandem *trnQ-UUG* genes were divided into two parts: one located near *rps16* and *chlB* genes, the other located near *psbK* and *psbI* genes. In *C. lanceolata*, three copies of *trnQ-UUG* gene were identified, with two copies of the same orientation and another with the opposite orientation (Fig. 2). Here, we also speculate one possible evolutionary process for the formation of the three copies of *trnQ-UUG*. We suppose that two copies of *trnQ-UUG* were located between LCB 1 and 12 in A25. From A25 to *C. lanceolata*, one inversion happened between LCB 12 and 29 (Fig. 3 the blue line). This inversion separates the two *trnQ-UUG* gene into two parts, making one copy of *trnQ-UUG* located in LCB1 and the other in LCB 12. Combined with one copy of *trnQ-UUG* located in LCB 2, finally, the three copies of *trnQ-UUG* were located in LCB 2, 1, and 12 respectively. In conclusion, the *trnQ-UUG* gene first formed tandem repeats, then a rearrangement divided the tandem *trnQ-UUG* repeat into different parts, forming inverted or forward repeats locating at different regions.

All the species containing *trnQ*-repeats (including tandem repeats and inverted repeats) formed a monophyletic group, while Podocarpaceae and Araucariaceae composed the other (Fig. 2b). This dichotomy is exactly consistent with the phylogenetic relationship inferred from other gymnosperm phylogenetic studies^13^,^14^. Furthermore, the transition stage of *trnQ-UUG* also indicated *S. verticillata* as the basal of the Cupressaceae-Taxaceae-Cephalotaxacea clade. Therefore, *trnQ-UUG* repeats in cupressophytes may be considered as informative markers for conifer phylogeny.

### *AccD* has been functionally transferred from the chloroplast to the nucleus

**Figure 4:**
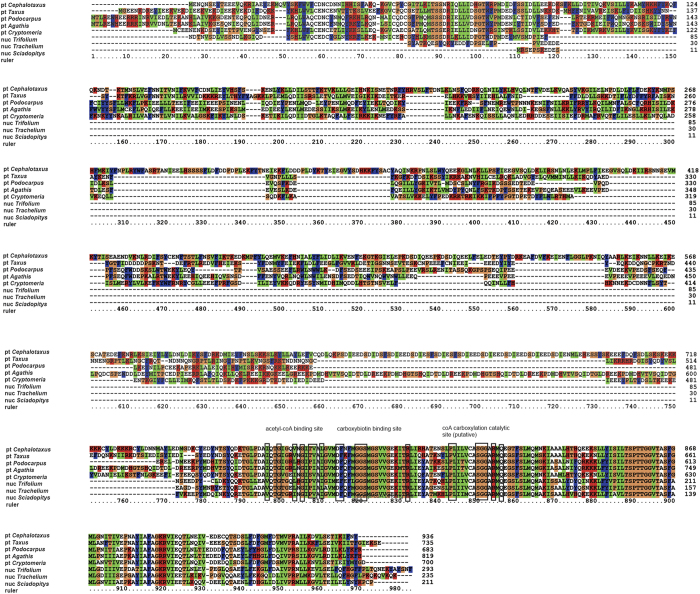
Multiple alignment of the predicted nuclear *accD* from *Trifolium repens, Trachelium caeruleum, Sciadopitys verticillata* and plastidic *accD* from *Cephalotaxus oliveri, Taxus mairei, Podocarpus lambertii, Agathis dammara* and *Cryptomeria japonica*. The putative acetyl-CoA binding site, the carboxybiotin-binding site and the CoA-carboxylation catalytic site were shown in black box. The *T. repens* and *T. caeruleum accD* gene sequence only contained the 5′-terminal region of the *accD* gene (the position 513–805 for *T. repens* and the position 96–330 for *T. caeruleum* of the entire transcript).

**Figure 5:**
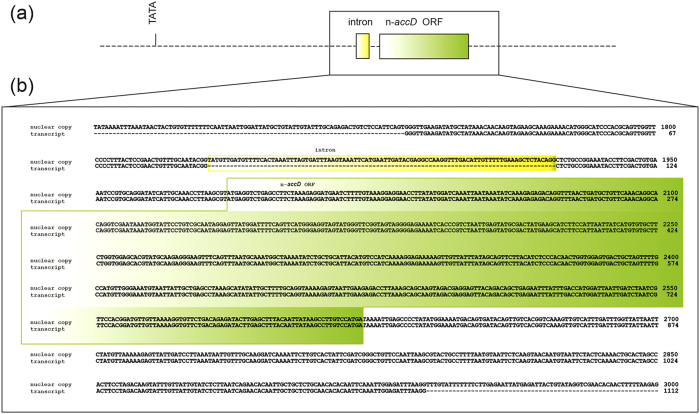
The gene structure of the nuclear *accD* sequence from *Sciadopitys verticillata.* (**a**) The coding region and intron are indicated by green and yellow boxes, respectively. Dashed lines denote noncoding regions. (**b**) Nucleotide sequence alignment of nuclear and transcript copies. The alignment region corresponds to the black box section shown in (**a**). Intron in the 5′ UTR and nuclear *accD* open reading frame are indicated with yellow and green boxes, respectively.

The complete cp genome sequence of *S. verticillata* showed that its *accD* gene was completely lost. A partial transcript containing high sequence identity to plastid *accD* was assembled using two *S. verticillata* transcriptome databases. Then the entire transcript of the *accD* sequence was obtained by using RACE. This transcript encodes a protein of 212 amino acids, compared with the approximately 680–1070 amino acids encoded by the *accD* gene in the chloroplast genome of other cupressophytes (Fig. 4). The 212 amino acids in this transcript are 84% identity to the *accD* carboxylase domain encoded by other cupressophyte plastid-*accD* genes. From Fig. 4, we infer that residues 25–212 (position 787–983 in the alignment) in the *accD* origin from plastid. The sequences located at 1985 to 1 bp upstream from the translation start site and 1 to 1066 downstream of the stop codon were obtained by anchored PCR (Fig. 5). Predicted eukaryotic TATA box were identified −1599 bp 5′ of the translation start site of the gene. The flanking region of *accD* sequence showed no plastid sequence similarity. In addition, blastn of this sequence to the nucleotide database in GenBank returns no match, suggesting that it is likely originated from nuclear or mitochondria. The flanking regions of the *accD* containing sequence also had no significant hits with blast to *Cycas taitungensis* mitochondria genome, the only one reported mitochondria genome in gymnosperm, which indicated that *accD* is more likely located in nucleus. Five different softwares (BaCelLO, MultiLoc, Predotar, Protein Prowler, and TargetP) were used to predict the target peptide-encoding sequences of nuclear *accD* (n-*accD*) sequence. None of the softwares identified any target peptide-encoding sequences to chloroplast. The BaCelLO, MultiLoc and Protein Prowler predicted target peptide-encoding sequences to cytoplasm, while Predotar and TargetP predicted to elsewhere (Supplementary Table S3). The n-*accD* gene was also amplified from genomic DNA. A single intron of 93 bp is present at the 5′ UTR region, which is located at −65 and −64 bp upstream of the ORF (Fig. 5). The nucleus copy and transcript of n-*accD* sequences showed 100% identity. Thus no RNA editing sites were found in the n-*accD* copy (Fig. 5).

### Phylogenetic distribution of *accD* loss in gymnosperm

**Figure 6:**
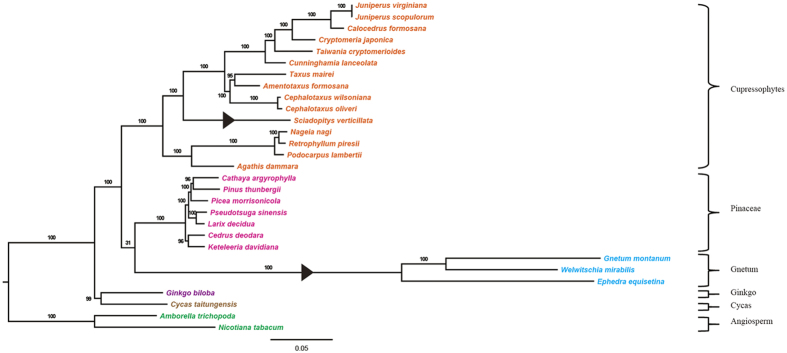
Maximum likelihood tree of 29 taxa from 59 plastid genes. Bootstrap values are shown along branches. Triangles on nodes indicate loss of *accD* from plastome.

In order to determine the phylogenetic distribution of *accD* loss in gymnosperm, we constructed a maximum likelihood tree inferred from the concatenated 59 cpDNA genes (50555 nucleotides) with *Amborella trichopoda* and *Nicotiana tabacum* as outgroup (Fig. 6). In this topology, Cycas and Ginkgo were grouped together; then Gnetales was clustered with Pinaceae (the gnepines hypothesis). For cupressophytes, the two families, Araucariaceae and Podocarpaceae, diverged first, and then Sciadopityaceae was strongly supported to be sister to a well-supported clade containing Cupressaceae, Taxaceae and Cephalotaxaceae. At last, Taxaceae and Cephalotaxaceae formed a monophyly and then clustered with Cupressaceae. The phylogenetic position of Sciadopityaceae in our study was consistent with previous molecular phylogenetic studies^12^,^13^. A functional plastid *accD* still exists in Cycas, Ginkgo and other conifers. However, for Sciadopityacea and Gnetales, *accD* has been lost from their plastomes. This topology (Fig. 6) indicates two independent losses of *accD* from their plastomes. However, for Gnetales, no studies have been performed to demonstrate if there is a functional copy in the nucleus.

### The origin of nuclear *accD* gene

**Figure 7:**
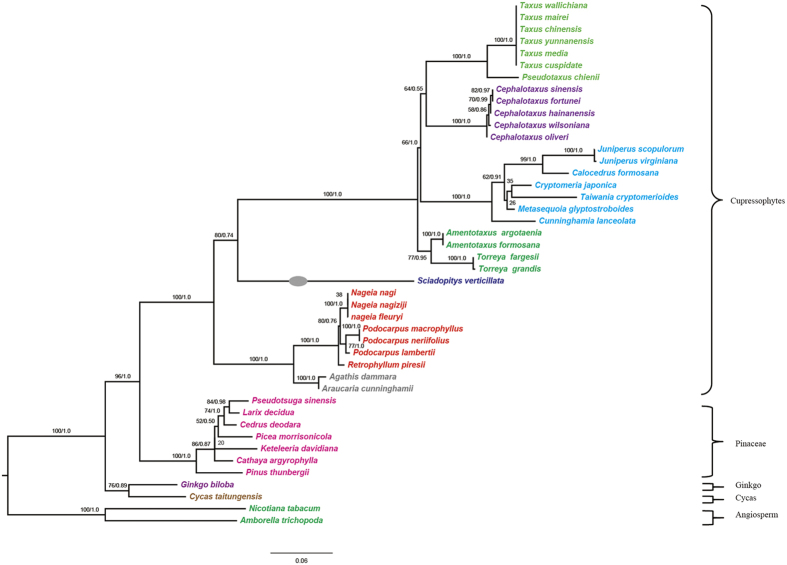
Phylogenetic relationship of *Sciadopitys verticillata* nuclear *accD* sequences to chloroplast *accD* sequences from other gymnosperms. The tree was constructed using Maximum likelihood and Bayes methods. Bootstrap and posterior probability values are shown along branches. Gray oval on node indicates the position of *S. verticillata* in the topology.

Phylogenetic analysis of the n-*accD* transcript in *S. verticillata* and plastid-encoded counterparts from 43 other plants was used to infer the origin of n-*accD* in *S. verticillata*, with *A. trichopoda* and *N. tabacum* as outgroup (Fig. 7). The results showed that n-*accD* transcript of *S. verticillata* was located in the basal clade of the Cephalotaxaceae, Taxaceae and Cupressaceae plastid copies, which was consistent with the phylogeny tree constructed by 59 cpDNA genes. Considering that other cupressophytes (Araucariaceae, Podocarpaceae, Cupressaceae, Taxaceae and Cephalotaxaceae) contain an intact copy of plastid *accD* gene, we suggest that *accD* has been functionally transferred to the nucleus independently near the time of divergence of the Cephalotaxaceae-Taxaceae-Cupressaceae and Sciadopityaceae.

## Disscussion

In this study, we have determined the complete chloroplast genome sequence of *S. verticillata*, which is the only one species of Sciadopityaceae. Like other sequenced cp genomes of cupressophytes^4^,^5^, the *S. verticillata* cp genome has no IRs (Fig. 1). It is most similar to the cp genome of *Amentotaxus formosana*, with 8 inversions involved in (Fig. 2b). When compared with *C. oliveri, S. verticillata* had 10 specific rearrangements (Fig. 2b). The large number of rearrangements between *S. verticillata* and other cupressophytes cp genome indicate that *S. verticillata* has a distinctive cp genome structure among cupressophytes. Previous researches have shown that the presence of IRs can stabilize cp genome organization, while their absence often leads to rearrangements^4^,^32^,^33^. The extensively rearranged cp genome of *S. verticillata* without IRs supports this suggestion.

We have proposed one mechanism of the formation of small inverted repeats. The tandem repeat of *trnQ-UUG* occurred first by a process of slipped-strand mispairing in *S. verticilla*, and the subsequent inversion resulted in the inverted repeat (Fig. 3). Tandem repeats may form totally by chance, for example, as a result of replication slippage^34^. For the formation of inverted repeat, Knox has proposed three mechanisms: coincidental similarities, transposed copies, and duplications at inversion endpoints^35^. Coincidental similarities usually lead to a half dozen of inverted repeats with moderate-to-high sequence similarity in a typical plastome^35^. The dispersed repeats generated by duplicative transposition of cp DNA would not be inverted relative to the source region, but subsequent inversion put these copies in inverted orientation^35^,^36^. Some inversions raise duplications that produce inverted fragments at both junctions^35^,^37^. Hence it is more difficult to form inverted repeat than tandem repeat. There are two types of genome organizations around *trnQ-UUG* in the clade that contains *trnQ-UUG* inverted repeat (Supplementary Fig. S1). One type is that one copy of *trnQ-UUG* is located near *chlB* and *trnT-UGU*, and the other situated between *psbK* and *trnL-UAA*. The other type shows that one copy of *trnQ-UUG* is located near *chlB* and *psbK*, while the other is adjacent to *trnT-UGU* and *trnL-UAA*. However, the two types can transform into each other by the occurrence of an inversion located between the two inverted copies of *trnQ-UUG* (Supplementary Fig. S1). Similarly, a large 36-kb inversion among four *Juniperus* plastomes was also suggested to be caused by an approximately 250 bp-IR containing *trnQ-UUG*^6^. The relative fixed location of the two *trnQ-UUG* genes indicates that the inverted repeat in Taxaceae, Cephalotaxaceae, and Cupressaceae originates from a common ancestor. We thus suggest that the inverted repeat is originated from tandem repeats in Sciadopityaceae. These inverted repeats can serve as a molecular basis of inversions, and inversions in turn promote the formation of inverted repeats^6^,^35^.

The *accD* gene encodes the β-carboxyl transferase subunit of ACCase. ACCase catalyzes the formation of malonyl-CoA from acetyl-CoA and is used in *de novo* fatty acid synthesis^26^. Plants have two forms of ACCase: the eukaryote form located in cytosol and the prokaryote form located in plastids^26^. The eukaryote-form ACCase is composed of a single multifunctional polypeptide, whereas the prokaryote-form is comprised of four subunits: the α-carboxyltransferase subunit (*accA*), the biotin carboxyl carrier (*accB*), the biotin carboxylase (*accC*), and the β-carboxyltransferase subunit (*accD*). *AccA, accB, accC* are all nucleus-encoded. In contrast, *accD* is encoded in the plastome. *AccD* is widely distributed in plants, even in some parasitic and non-photosynthetic plants^38^. In Campanulaceae and Fabaceae, *accD* has been functionally transferred to the nucleus^23^,^24^. In this study, we found that *accD* gene is lost in the plastome of Sciadopityaceae; and furthermore, the chloroplastic *accD* gene of *Sciadopity* have been transferred to the nucleus (Fig. 4).

The case of *accD* in Sciadopityaceae represents the third documented transfer for this gene from plastid to nucleus with the other two occuring in the unrelated angiosperm families Campanulaceae and Fabaceae. In *Trachelium caeruleum* (Campanulaceae) and *Trifolium repens* (Fabaceae), the nucleus (n)-*accD* transcripts encode 235 and 293 amino acids of plastid origin, respectively. Both in Campanulaceae and Fabaceae, the n-*accD* genes encode only the 3′-end region of the plastid gene. Consistently, the *S. verticilla* n-*accD* gene also only encodes the 3′-end region of *accD* gene (Fig. 4). These indicates that the C-terminus region of ACCD protein is the main functional domain. In the potato plastid *accD*, three functionally relevant sites have been identified: a putative acetyl-CoA binding site, a CoA-carboxylation catalytic site, and a carboxybiotin-binding site^39^. The three sites, clustering at the C-terminus of the protein, are present in the n-*accD* sequence in Campanulaceae, Fabaceae, and Sciadopityaceae (Fig. 4).

The major difference among the three transfers is that the Sciadopityaceae n-*accD* has no chloroplastic transit peptide sequence at the N-terminus of its transcript. In *T. repens* and *T. caeruleum*, the n-*accD* shares a transit peptide with *LPD2 and KASI*, respectively. More specifically, in *T. caeruleum*, a chloroplastic transit peptide was verified experimentally by showing that the product of n-*accD* was imported into the chloroplast^24^. For the acquisition of transit peptide, two different strategies were generally used. Some nucleus copies have acquired a novel transit peptide, such as the transfer of *rpl32* in *Thalictrum* and *Aquilegia*^22^; *infA* in multiple lineages of rosid^17^, and *rpoA* in moss *Physcomitrella patens*^40^. In contrast, other transferred genes acquired their transit peptide by transferring into a duplicate copy of a nuclear gene that has been already targeted to the plastid. For example, in *Bruguiera* and *Populus, rpl32* is fused to an existing nuclear gene (Cu-Zn superoxide dismutase)^20^,^21^; in Campanulaceae and Fabaceae, *accD* is fused to *KASI* and *LPD2*, respectively^23^,^24^. However, in Sciadopityaceae, we falied to identify the chloroplastic transit peptide sequence by using five softwares (Supplementary Table S3). Further studies are needed to explore the mechanism of function replacement of *accD* gene in Sciadopityaceae.

We have used two data sets to infer the phylogenetic position of Sciadopityaceae: the permutation of LCBs based on arrangements of cpDNA organization and 59-gene sequences. The two data sets yield the same result: Sciadopityaceae is sister to the clade comprising Taxaceae, Cephalotaxaceae, and Cupressaceae (Figs 2b and 6). Our result is in good agreement with previous molecular phylogenetic studies^13^,^41^. This further shows the usefulness of the cpDNA organization to resolve the phylogenetic relationships at the familial level and above. Rarrangements of cpDNA fragments have previously provided novel evidence for gnepines hypothesis^9^. Nonetheless, isomeric plastomes caused by sIR and homoplasious character should be treated cautiously when using genomic rearrangements in phylogenetic inferences^7^.

## Conclusions

We have determined the complete cp genome sequence of *S. verticillata*. The *S. verticillata* cp genome is highly divergent with a distinctive genome structure comparing with other cupressophyes. Our data suggest a molecular mechanism for the formation of small inverted repeat sequences in cupressophytes; that is rearrangements divide the tandem repeat into different parts, forming inverted or forward repeats locating at different regions. One unusual feature of the *S. verticilla* cp genome is the loss of *accD* gene. Examination of transcriptome database indicates that this gene has been transferred to the nucleus. The transfer time was estimated to be in the divergence of the Sciadopityaceae and Cephaloceae-Taxaceae-Cupressaceae. Moreover, phylogenetic relationships yielded by using 59-gene sequences and cp genome organizations both strongly support the placement of Sciadopityaceae as the sister of the clade containing Cephalotaxacea, Taxaceae, and Cupressaceae.

## Methods

### Plant material and chloroplast genome sequencing

*S. verticillata* is an evergreen conifer endemic to Japan. It is monoecious and wind pollinated reaching heights of 45 m, diameters of up to 2 m^42^. As an economically valuable tree, *S. verticillata* is often used in construction. The inheritance of chloroplast and mitochondria in *S. verticillata* is through paternal transmission^43^. *S. verticillata* is particularly valuable as the last member of the formerly widespread and more diverse conifer family, the Sciadopityaceae.

Young leaves of *S. verticillata* were collected from a single individual growing in Lushan Botanical Garden, Jiangxi Province. The materials used for RNA extraction was saved in RNAfixer (Bioteke Corporation, Beijing, China).Voucher specimens were deposited at the herbarium of Wuhan Botanical Garden, Chinese Academey of Science with the accession number of Lijiachenshanshan001. The genomic DNAs were isolated from the fresh leaves using the modified CTAB method. 1 μg DNA was sheared by Covaris M220 (Covaris, USA), yielding fragments of 500 bp in length. Paired-end libraries were constructed using NEBNext UltraTM DNA Library Prep Kit for Illumina (NEB, USA) according to the manufacturer’s instructions. Genomic DNA was sequenced on a single lane using the Illumina HiSeqTM 2500 platform (Illumina Inc., San Diego, CA). Approximately 3.14 GB of 150-bp paired-end raw reads were generated. FASTX-Toolkit (http://hannonlab.cshl.edu/fastx_toolkit/) was used to remove adaptor and low-quality reads. The clean reads were *de novo* assembled by velvet (V1.2.07)^44^, with the coverage cutoff value as 30 (-cov_cutoff 30). Among the 22 assembled contigs, 15 contigs were found to match the published conifers cpDNA sequence and used for complete genome finishing. The gaps were bridged by polymerase chain reaction (PCR) amplification based on the cp contig sequence. Overlapping regions of adjacent PCR products were set to at least 300 bp. PCR amplification was carried out in 50 μl volumes containing 2.5 ng of DNA template, 5 μl 10 × LA PCR Buffer II (Mg2 + Plus), 8 μl dNTP mixture (each 2.5 mM), 2.5 U of LA Taq (TaKaRa Bio Inc, Dalian, China), and 2 μl each of forward and reverse primers (10 μM). The thermo-cycling program was set as: 5 min at 95 °C (1 cycle); 30 s at 95 °C, 3 min at 62 °C (32 cycles); 20 min at 72 °C (1 cycle). Positive PCR amplicons were sequenced on ABI 3730 xl DNA Analyzer (Applied Biosystems, Foster City, CA, USA). At last, all the contigs and PCR amplification sequences were assembled into a complete chloroplast genome using Bioedit^45^.

### Genome annotation

The cp genome of *S. verticillata* was initially annotated by Dual Organellar GenoMe Annotator (DOGMA)^46^. The exact boundaries of each gene was determined by comparisons with homologous genes in other published gymnosperm cpDNAs. tRNA genes were further verified by two programs, ARAGORN^47^ and tRNAscan-SE^48^. The circular gene map was drawn by the software OGDRAW^49^.

### Identification and isolation of *accD* gene in the *S. verticillata* nuclear genome

Two *S. verticillata* transcriptome data (SRR065035, ERR364344) from 454 and illumina sequencing were downloaded from Sequence Read Archive (SRA) database (http://www.ncbi.nlm.nih.gov/Traces/sra/). Using *Nageia nagi accD* sequences as the query to blast against these two transcriptome dataset, 19 and 100 reads were identified for SRR065035 and ERR364344, respectively. All of these reads were then assembled into a approximately 1000 bp long sequence using Bioedit^45^. This sequence was first verified by PCR using appropriate primer pairs (Supplementary Table S4, naccDF and naccDR). The flanking sequence of this 1000 bp sequence was acquired by Anchored PCR^50^ using gene specific primers (Supplementary Table S4).

Total RNA was extracted using GREENspin Plus Plant RNA kit (ZoManBio, Beijing, China) and genomic DNA was removed using RNase-free DNase I (Takara, Dalian, China). cDNA templates were synthesized using Reverse Transcriptase M-MLV Kit (Takara Bio Inc., Dalian, China). Rapid amplification of cDNA ends (RACE) was conducted using SMART^TM^ RACE cDNA Amplification Kit (Clontech, Palo Alto, CA). The primers used for cloning 5′ and 3′ cDNA ends are the same as those used in Anchored PCR (Supplementary Table S4). All kits were used according to the manufacturers’ instructions.

Five softwares were used to identify transit peptides, BaCello^51^, MultiLoc^52^, Predotar^53^, Protein Prowler^54^ and TargetP^55^.

### Computational methods and phylogenetic analysis

The Perl script MISA (http://pgrc.ipk-gatersleben.de/misa/) was used to identify simple sequence repeats (SSR) in *S. verticillata* cp genome. The thresholds for SSR search was defined as ten repeat units for mono-nucleotides, five repeat units for di-nucleotides, and four repeat units for tri-, tetra-, penta-, and hexa-nucleotides. Tandem repeats were analyzed using Tandem Repeats Finder (http://tandem.bu.edu/trf/trf.submit.options.html) with the basic model. All of the repeats found were manually verified, and the nested or redundant results were removed.

Phylogenetic analyses were performed on three data sets. The first contained 15 cupressophyte chloroplast sequences (Supplementary Table S5). The second data set included 15 cupressophyte species from the first data sets, 12 other gymnosperm and 2 angiosperms whose plastid genomes were completely sequenced (Supplementary Table S5). The third data set included 25 *accD* sequences extracted from the second data set (excluding three Gnetales species and *Sciadopity* whose *accD* gene was lost), 18 *accD* sequences reported in previous research and n-*accD* in *S. verticillata* (Supplementary Table S6).

For the first data set, whole-genome alignment was performed using progressive Mauve implemented in MAUVE v.2.3.1^56^. Thirty-three LCBs were identified. MGR 2.0.1 (http://grimm.ucsd.edu/MGR/pubs.html) was used to build the phylogenetic trees and the process of rearrangement based on matrices of LCBs with the model of unichromosomal circular reversal distance.

Fifty-five common protein-coding and four rRNA genes were extracted from the cp genomes of the second data set (Supplementary Table S5). Each gene was aligned using the MUSCLE program implemented in MEGA 6. The aligned sequences were concatenated into a 59-gene data set and then used for reconstructing the gymnosperm phylogeny. Maximum likelihood (ML) tree was performed by RaxML v8.1.x^57^ with a GTRCAT substitution model as suggested (see RAxML manual). Clade supports were identified with 100 bootstrap replicates.

Forty-four *accD* sequences from the third data set were aligned using MUSCLE as implemented in MEGA 6. ML and Bayes inference (BI) were used to construct the phylogeny relationship of this forty-four *accD* sequences. ML trees were conducted with GTRCAT model using RaxML v8.1.x. Supports for nodes of trees were evaluated by 100 bootstrap replications. Bayesian inference (BI) trees were constructed by MrBayes v.3.1.2^58^ with the GTR + I + G model selected by Modeltest 3.7. Four independent Markov chain Monte Carlo chains were run with 1000000 generations. The first 25% of the sampled trees were removed as burn in. The remaining trees were used to construct a 50% majority-rule consensus tree.

### Data availability

All sequencing data produced in the present work have been submitted to Genbank under the accession KT601208-KT601211 and Sequence Read Archive with the accession of SRP067546.

## Additional Information

**How to cite this article**: Li, J. *et al*. Evolution of short inverted repeat in cupressophytes, transfer of *accD* to nucleus in *Sciadopitys verticillata* and phylogenetic position of Sciadopityaceae. *Sci. Rep.* **6**, 20934; doi: 10.1038/srep20934 (2016).

## Supplementary Material

Supplementary Information

## Figures and Tables

**Table 1:** Comparison of chloroplast genomic characteristics between the four cupressophytes.

Taxa	*Amentotaxus argotaenia* (KR780582)	*Sciadopitys verticillata* (KT601210)	*Cephalotaxus wilsoniana* (NC_016063)	*Taxus mairei* (NC_020321)
Genome size (bp)	136657	138284	136196	127665
Protein-coding genes (%)	55.08	55.26	56.78	50.12
tRNA genes (%)	1.82	1.80	1.61	1.57
rRNA genes (%)	3.37	3.4	3.27	3.58
Introns (%)	8.78	9.12	9.15	10.34
Spacers (%)	30.95	30.4	29.19	34.37
Gene density (no. of gene/Kb)	0.88	0.87	0.85	0.88
GC content (%)				
Genome	35.85	35.42	35.08	34.72
Protein-coding genes	36.90	36.35	36.04	36.76
tRNA genes	53.31	52.79	52.94	52.36
rRNA genes	52.99	52.45	52.14	52.57
Introns	36.10	35.93	35.08	34.63
Spacers	31.03	30.65	30.29	29.97
