# Supplementary material for: Evolution of short inverted repeat in cupressophytes, transfer of accD to nucleus in Sciadopitys verticillata and phylogenetic position of Sciadopityaceae
Source: Sci Rep. 2016 Feb 11;6:20934. doi: 10.1038/srep20934 (PMC4750060; doi:10.1038/srep20934)
Supplement: Supplementary Information [file srep20934-s1.pdf]

Evolution of short inverted repeat in cupressophytes, transfer of *accD* to nucleus in *Sciadopitys verticillata* and phylogenetic position of Sciadopityaceae

Jia Li[1, 2,†], Lei Gao[1,†], Shanshan Chen[1, 2], Ke Tao[1, 2], Yingjuan Su[3, 4,*], and Ting Wang[1, 5,*]

[1] CAS Key Laboratory of Plant Germplasm Enhancement and Specialty Agriculture, Wuhan Botanical Garden, Chinese Academy of Sciences, Wuhan, Hubei, China, [2] University of Chinese Academy of Sciences, Beijing, China, [3] State Key Laboratory of Biocontrol, School of Life Sciences, Sun Yat-sen University, Guangzhou, Guangdong, China, [4] Institute for Technology Research and Innovation of Sun Yat-sen University, Zhuhai, Guangdong, China, and [5] College of Life Sciences, South China Agricultural University, Guangzhou, Guangdong, China.

[†] These authors contributed equally to this paper.

[*]Correspondence: Ting Wang, Tel: +86-27-87510677. Fax: +86-27-87510251. E-mail address: tingwang@wbgcas.cn;

Yingjuan Su, Tel: +86-20-84111939. Fax: +86-20-84036215. E-mail: suyj@mail.sysu.edu.cn

Supplementary Table S1. Distribution of simple sequence repeats in *Sciadopitys verticillata* chloroplast genome.

SSR nr.	SSR	size	Start-end	Location
1	(G)12	12	7929--7940	*chlB/trnN-GUU* (IGS)
2	(A)21	21	9596--9616	*rrn4.5-rrn23* (IGS)
3	(TTTG)4	16	10797--10812	*rrn23*
4	(T)11	11	14615--14625	*trnI-GAU/rrn16* (IGS)
5	(AT)5	10	15300--15309	*trnI-GAU/rrn16* (IGS)
6	(AG)5	10	15702--15711	*trnI-GAU/rrn16* (IGS)
7	(A)13	13	19017--19029	*trnV-GAC/rps12 exon2* (IGS)
8	(A)19	19	20215--20233	*rps12 intron*
9	(AT)5	10	20288--20297	*rps12 intron*
10	(A)11	11	35682--35692	*rpl2 intron*
11	(A)13	13	38632--38644	*rpl16 intron*
12	(A)18	18	40655--40672	*rps8/infA* (IGS)
13	(T)10	10	41474--41483	*rpl36/rps11* (IGS)
14	(AGA)5	15	45759--45773	*petB intron*
15	(AT)21	42	46417--46458	*psbH/psbN* (IGS)
16	(AT)9	18	47048--47065	*psbT/atpF* (IGS)
17	(A)13	13	48467--48479	*atpF/atpH* (IGS)
18	(T)13	13	51081--51093	*rps2/rpoC2* (IGS)
19	(T)10	10	51289--51298	*rpoC2*
20	(T)10	10	51328--51337	*rpoC2*
21	(T)10	10	53294--53303	*rpoC2*
22	(A)10	10	57791--57800	*petL/psbE* (IGS)
23	(T)10	10	61455--61464	*petA/cemA* (IGS)
24	(A)11	11	61557--61567	*petA/cemA* (IGS)
25	(A)11	11	61584--61594	*petA/cemA* (IGS)
26	(T)10	10	62061--62070	*cemA*
27	(A)10	10	62908--62917	*cemA/ycf4* (IGS)
28	(AT)9	18	65597--65614	*trnE-UUC/trnT-GGU* (IGS)
29	(AT)18	36	66522--66557	*trnT-GGU/psbD* (IGS)
30	(T)16	16	69481--69496	*psbC/trnS-UGA* (IGS)
31	(A)10	10	69868--69877	*trnS-UGA/psbZ* (IGS)
32	(T)13	13	70583--70595	*trnG-GCC/trnfM-CAU* (IGS)
33	(A)10	10	70817--70826	*trnfM-CAU/rps14* (IGS)
34	(G)12	12	79121--79132	*trnS-GGA/rps4* (IGS)
35	(G)14	14	80451--80464	*trnT-UGU/trnL-UAA* (IGS)
36	(AT)19	38	81507--81544	*trnF-GAA/ndhJ* (*IGS)*
37	(T)11	11	81642--81652	*trnF-GAA/ndhJ* (IGS)
38	(ATT)4	12	85058--85069	*trnV-UAC intron*
39	(AT)9	18	85631--85648	*trnV-UAC/trnM-CAU* (IGS)
40	(T)10	10	86093--86102	*trnM-CAU/atpB* (IGS)
41	(TA)15	30	88468--88497	*atpE/rbcL* (*IGS)*

42	(T)10	10	90718--90727	*trnR-CCG/trnR-UCU* (IGS)
43	(A)11	11	90863--90873	*trnR-UCU/trnG-UCC* (IGS)
44	(A)12	12	101075--101086	*ycf1*
45	(A)10	10	102814--102823	*ycf1*
46	(AGA)7	21	106389--106409	*ycf1*
47	(TGA)8	24	107862--107885	*ycf1*
48	(A)13	13	122238--122250	*rpoB*
49	(T)16	16	130248--130263	*rps12 exon1*
50	(TTC)4	12	130571--130582	*rps12 exon1/clpP* (IGS)
51	(T)10	10	130748--130757	*clpP*
52	(A)11	11	135094--135104	*atpA*
53	(TA)8	16	137825--137840	*trnC-GCA/trnI-CAU* (IGS)

Note：IGS, intergenic spacers.

Supplementary Table S2. Distribution of tandem repeats in *Sciadopitys verticillata* chloroplast genome.

No.	Repeat length	Consensus size copy number	Start-end	location
1	359	53 × 6.8	14932--15290	*trnI-GAU*/*rrn16* (IGS)
2	272	134× 2	15306--15577	*trnI-GAU*/*rrn16* (IGS)
3	146	43 × 3.4	17637--17782	*rrn16*/ *trnV-GAC* (IGS)
4	77	39 × 2	28223--28299	*ycf2*
5	133	66 × 2	29468--29600	*ycf2*
6	66	33×2	29791--29856	*ycf2*
7	189	36× 5.3	30734--30922	*ycf2*
8	314	75 × 4.2	31264--31577	*ycf2*
9	620	33 × 18.2	31975--32594	*ycf2*
10	389	179 ×2.2	33730--34118	*ycf2*/ *trnI-CAU* (IGS)
11	126	66 × 1.9	86082--86207	*trnM-CAU*/*atpB* (IGS)
12	207	73 × 2.8	94306--94512	*trnQ-UUG*
13	524	170 × 3.1	98374--98897	*rpl32*/*ycf1* (IGS)
14	116	57 × 2	99956--100071	*ycf1*
15	156	42 × 3.7	101424--101579	*ycf1*
16	273	39 × 6.9	101929--102201	*ycf1*
17	181	60 × 3	104785--104965	*ycf1*
18	137	63 × 2.2	125661--125797	*rpoC1* intron

Note：IGS, intergenic spacers.

Supplementary Table S3. Prediction of the presence of a transit peptide-encoding sequence in the nuclear *accD* transcript of *Sciadopitys verticilla* using five different softwares.

	ptTP score	mTP score	Secretory pathway	Elsewhere	cytoplasm
BaCelLO					**yes**
MultiLoc					**0.89**
Predotar	0.00	0.01		**0.99**	0.01
Protein Prowler	0.100	0.100			**0.650**
TargetP	0.058	0.179	0.087	**0.917**	

Supplementary Table S4. Primers used in this study

Primer name	direction	Primer Sequence (5'-3')
naccDF	F	ACTGTCTCCATTCAGTGGGTT
naccDR	R	CGCTTAATTGGAACAGCCCGATC
GSP3	R	TCCTCTTTAGAAGGCTCAGACC
GSP1	R	TTTATTCGACCTGTGCCTGT
GSP4	F	ACCTTAAAGCAGCAAGTAGACG
GSP2	F	CTTACATCTCCCACAACTGGT
GSP5R	R	GTCGAAGGTATTTCCGGCAGA
GSP6F	F	CCACGGATGTTGTTAAAAGGTGT
SP1L	R	TCGCATACTCAATTAGACGGGTGA
SP1R	F	TGACTGCTAGTTTTGCCATGTTGGG

Supplementary Table S5. Plant materials and GenBank accession numbers of the 29 species whose plastid genomes were completely sequenced.

Taxa	Family	Genus	Species	GenBank Accession No.
Gymnosperm	Cephalotaxaceae	*Cephalotaxus*	*Cephalotaxus wilsoniana*	NC_016063
	Cephalotaxaceae	*Cephalotaxus*	*Cephalotaxus oliveri*	NC_021110
	Cupressaceae	*Cryptomeria*	*Cryptomeria japonica*	NC_010548
	Cupressaceae	*Taiwania*	*Taiwania cryptomerioides*	NC_016065
	Cupressaceae	*Calocedrus*	*Calocedrus formosana*	NC_023121
	Cupressaceae	*Cunninghamia*	*Cunninghamia lanceolata*	NC_021437
	Cupressaceae	*Juniperus*	*Juniperus virginiana*	NC_024024
	Cupressaceae	*Juniperus*	*Juniperus scopulorum*	NC_024023
	Araucariaceae	*Agathis*	*Agathis dammara*	NC_023119
	Podocarpaceae	*Agathis*	*Nageia nagi*	NC_023120
	Podocarpaceae	*Podocarpus*	*Podocarpus lambertii*	NC_023805
	Podocarpaceae	*Retrophyllum*	*Retrophyllum piresii*	NC_024827
	Taxaceae	*Taxus*	*Taxus mairei*	NC_020321
	Taxaceae	*Amentotaxus*	*Amentotaxus formosana*	NC_024945
	Sciadopityaceae	*Sciadopitys*	*Sciadopitys verticillata*	KT601210
	Cycadaceae	*Cycas*	*Cycas taitungensis*	NC_009618
	Ginkgoaceae	*Ginkgo*	*Ginkgo biloba*	NC_016986
	Ephedraceae	*Ephedra*	*Ephedra equisetina*	NC_011954
	Gnetaceae	*Gnetum*	*Gnetum montanum*	NC_021438
	Welwitschiaceae	*Welwitschia*	*Welwitschia mirabilis*	NC_010654
	Pinaceae	*Cathaya*	*Cathaya argyrophylla*	NC_014589
	Pinaceae	*Cedrus*	*Cedrus deodara*	NC_014575
	Pinaceae	*Keteleeria*	*Keteleeria davidiana*	NC_011930
	Pinaceae	*Larix*	*Larix decidua*	NC_016058
	Pinaceae	*Picea*	*Picea morrisonicola*	NC_016069
	Pinaceae	*Pinus*	*Pinus thunbergii*	NC_001631
	Pinaceae	*Pseudotsuga*	*Pseudotsuga sinensis*	NC_016064
Angiosperm	Solanaceae	*Nicotiana*	*Nicotiana tabacum*	NC_001879
	Amborellaceae	*Amborella*	*Amborella trichopoda*	NC_005086

Supplementary Table S6. GenBank accession numbers of the 18 *accD* sequences reported in previous research.

Family	Genus	Species	GenBank Accession No.
Taxaceae	*Taxus*	*Taxus media*	KT030780
		Taxus wallichiana	KT030784
		Taxus chinensis	KT030782
		Taxus cuspidate	KT030781
		Taxus yunnanensis	KT030783
	Pseudotaxus	*Pseudotaxus chienii*	KT030788
	Torreya	*Torreya fargesii*	KT030785
		Torreya grandis	KT030786
	Amentotaxus	*Amentotaxus argotaenia*	KT030787
Cephalotaxaceae	*Cephalotaxus*	*Cephalotaxus hainanensis*	KT030790
		Cephalotaxus sinensis	KT030789
		Cephalotaxus fortunei	KT030791
Podocarpaceae	*Podocarpus*	*Podocarpus macrophyllus*	KT030792
		Podocarpus neriifolius	KT030793
	Nageia	*Nageia nagi*	KT030794
		Nageia fleuryi	KT030795
Araucariaceae	*Araucaria*	*Araucaria cunninghamii*	KT030797
Taxodiaceae	*Metasequoia*	*Metasequoia glyptostroboides*	KT030796

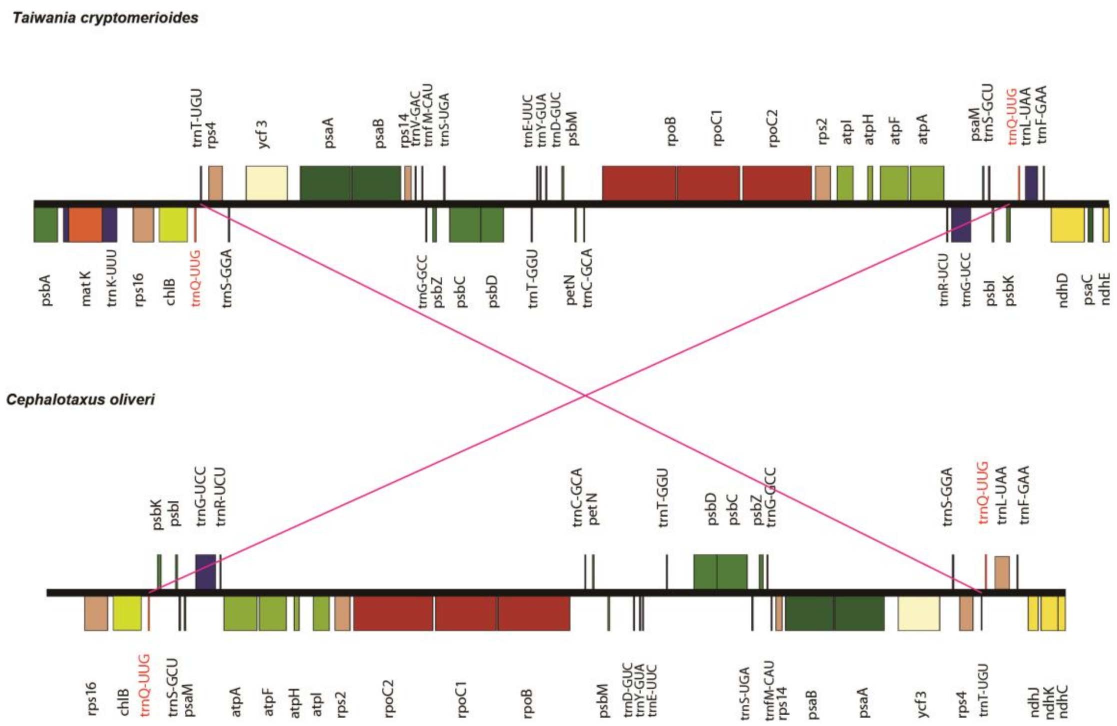

Supplementary Figure S1. The location of two copies of *trnQ-UUG* in cupressophytes chloroplast genome. One type of the genome organization around *trnQ-UUG* was represented by *Taiwania cryptomerioides*, while the other was represented by *Cephalotaxus oliveri*. The inversion between two plastomes was linked by carmine line.
